# Oral microbiota in an aging Swedish population with high dental disease burden: an observational registry-based study

**DOI:** 10.3389/froh.2025.1709163

**Published:** 2026-01-06

**Authors:** Anders Esberg, Simon Haworth, Ingegerd Johansson

**Affiliations:** 1 Department of Odontology, Umeå University, Umeå, Sweden; 2 Bristol Dental School, University of Bristol, Bristol, United Kingdom

**Keywords:** aging, oral microbiota, caries, periodontitis, SIMPLER

## Abstract

**Introduction:**

The global population is aging. Although aging populations experience a high burden of dental and systemic diseases, few studies have described the oral microbiota in aging population-based cohorts. This observational, registry-based study aimed to characterize the diversity and composition of the oral microbiota in 1,093 aging Swedes—aged 54–84 years at inclusion—and evaluate associations with host traits, as well as prospective measures of caries and periodontal status.

**Methods:**

Saliva microbiota was characterized using complete 16S rRNA gene sequencing, and dental data were obtained from primary care dental records. Partial least squares regression was used to identify species associated with variation in age, number of teeth, total number of sequence reads, caries, and periodontal status. Follow-up analyses were conducted using two-part regression models with covariate adjustments.

**Results:**

The oral microbiota remained highly diverse in the aging population without major shifts within this age frame. Carriage of hitherto unfamiliar yet well-documented disease-associated species was found to be associated with metrics of active disease but not lifelong measures, such as the common decayed, filled, and missing surfaces index.

**Conclusion:**

These results underscore methodological considerations, including the importance of read number adjustments beyond using relative abundances, and the importance of carefully selecting metrics for oral disease in aging individuals.

## 1 Introduction

Human microbiomes, including the oral microbiome, have attracted considerable attention for their dynamic interplay with host health and disease. The microbiome in the mouth is among the most complex and diverse in man with known impacts from host and exposure factors like genetics, diet, oral hygiene, and medications. In general, the oral microbiome is diverse and in balance with the host, whereas reduced diversity (dysbiosis) is a hallmark of oral diseases and suggestive of systemic diseases (1–3).

The oral microbiome undergoes changes through the life course with initiation and maturation in early life (4, 5), followed by fluctuations in childhood and young adults (6, 7). Thereafter, the oral microbiome is largely stable unless major events occur (8). Age-related changes in diet, oral hygiene, saliva secretion, and oral and systemic diseases and treatments may represent such major changes with potential effects on the taxonomic profile and diversity of the oral microbiota. Previous studies have substantiated the role of *Streptococcus mutans* and *Scardovia wiggsiae* in caries etiology (9) and *Aggregatibacter actinomycetemcomitans, Filifactor alocis, Porphyromonas gingivalis, Tannerella forsythia, Treponema denticola,* and *Treponema socranskii* in periodontitis etiology (10). However, their relative contributions in older adults may differ from that observed in younger adults. Few studies have described the oral microbiome profile and diversity in the aging population in relation to oral or general disease, and those available are based on small sample sizes and often focus on frail groups (11–13). Given the aging global population and the well-known age-related susceptibility to oral and systemic diseases, there is a requirement for population-based exploratory studies investigating the composition and function of the oral microbiota in aging adults.

The primary objective of this study was to characterize the diversity and composition of the saliva microbiome in a group of free-living aging and predominantly male individuals in Sweden. In addition, associations between microbiota characteristics and host demographics and dental status were explored. To achieve this, saliva DNA from the population-based Swedish Infrastructure for Medical Population-Based Life-Course and Environmental Research (SIMPLER) infrastructure (https://www.simpler4health.se/about-us/) was utilized for full 16S rRNA gene sequencing.

## 2 Methods

### 2.1 The SIMPLER infrastructure

**Figure 1:**
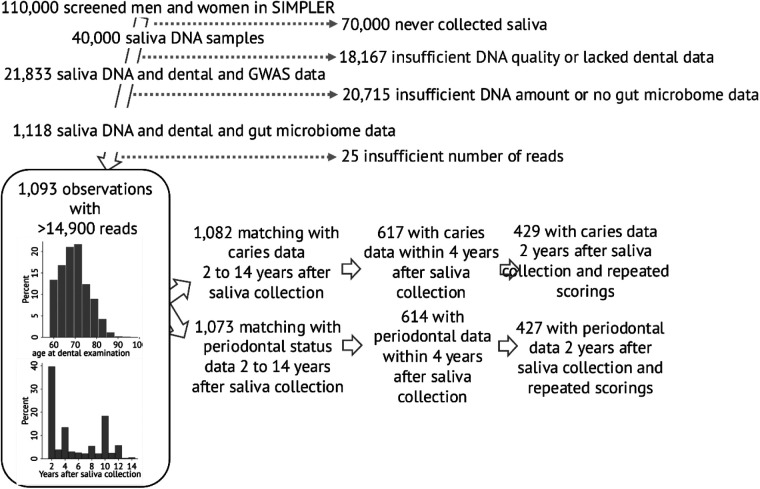
Flow diagram of cohort selection and bar graphs showing distribution of age at saliva collection and number of years between saliva collection and the first available dental examination. The number of participants with dental outcome data 2 years after saliva collection declines as their saliva-to-examination interval increases, reaching *n* = 424 and *n* = 384 for caries and *n* = 420 and *n* = 366 for periodontal status at 5- and 10-year differences, respectively.

The SIMPLER is a framework that compiles medical, genetic, dietary, and other lifestyle data on nearly 110,000 aging individuals in Sweden (https://www.simpler4health.se/about-us/). Participants who had donated saliva for DNA extraction were eligible (*n* = 40,000). Individuals lacking dental data or with insufficient DNA quality or quantity were excluded (Figure 1). For subsequent analysis, participants without information on gut microbiota or genome-wide association study (GWAS) data were excluded (Figure 1). After applying these inclusion and exclusion criteria, 1,118 participants remained, drawn from the Västerås-based Cohort of Swedish Men (COSM) and Swedish Mammography Cohort (SMC) sub-cohorts within SIMPLER, with a predominance of men from the COSM group. Besides providing saliva samples between 2006 and 2008, these participants took part in a health examination between 2010 and 2019, during which blood, urine, feces, and fat biopsy samples were collected. Additional exclusions (*n* = 25) were done due to insufficient number of sequencing reads, leaving 1,093 participants in the final study group (Figure 1).

### 2.2 Dental status from the SKaPa register

The SKaPa register is a national quality resource that compiles dental charting data from all public dental care clinics and some major private dental care chains in Sweden. It has collected information since 2008 on approximately 7.8 million patients and repeated visits (https://www.skapareg.se/other-language/).

Information on caries status was retrieved per tooth surface as sound (D0), caries in the enamel (D1), caries in the dentin (D2), and caries well into the dentin with a cavity (D3), restoration, and missing, with cause of missing if recorded. From this information, the number of caries-free surfaces (D0) and manifest caries lesions (D3) was scored. Further, an accumulated measure of caries experience (the sum of decayed, filled, and missing surfaces, DMFS) was used in confirmatory sensitivity analyses to address its previously reported insensitivity in aging populations (14). Third molars were excluded. For missing teeth and tooth-covering restorations, five surfaces were imputed as caries-affected for molar and premolar teeth, and four surfaces for canines and incisors.

Similarly, information on periodontal pocket probing depth (PPD) per six probing sites was retrieved and categorized as P0 (≤3 mm PPD), P1 (4 mm PPD), P2 (5 mm PPD), and P3 (≥6 mm PPD). In addition, data were collection on whether a periodontitis diagnosis (diagnosis code 3043) was assigned and if any periodontitis-associated treatment had been performed. Mean PPD, the total number of P3 pockets, and if a participant had at least two teeth with a probing pocket ≥6 mm or not were used as proxies for periodontal status. For data validation, it was confirmed that all participants with at least two teeth with a probing pocket ≥6 mm either had a reported periodontitis diagnosis or had undergone periodontitis-related treatment.

Systematic compilation of dental record data in the SKaPa register began in 2010. Depending on when a dental clinic joined the register or when a participant became affiliated with a participating clinic, the dental data closest to the time of saliva donation were available between 2010 and 2020, corresponding to 2–14 years after saliva collection. For a subgroup, follow-up recordings were available for up to 10 years after the first registered visit. The distribution of the shortest time gap between saliva donation and dental data is provided in Figure 1. For this study, we used either all data from the first available examination (*n* = 1,082 for caries and 1,073 for periodontal traits) or data with a 2-year or up to 4-year gap after saliva sampling for cross-sectional association analyses (Figure 1).

### 2.3 Microbiota characterization

#### 2.3.1 Saliva collection and DNA extraction

To obtain saliva DNA, Oragene OG-500 saliva collection kits (DNA Genotek Inc., Ottawa, ON, Canada) were sent by mail to the COSM/SMC participants. The participants were instructed to refrain from eating, drinking, smoking, or chewing gum for 30 min prior to the collection, and to actively spit saliva into the collection tube until the amount reached 2 mL. The tube was then capped, labeled, and returned by mail. The collection tube contained preservatives to protect DNA at room temperature (https://www.dnagenotek.com/row/products/collection-human/oragene-dna/500-series/OG-500). Upon return, the saliva samples were stored in −80 °C freezers at the SIMPLER biobank until transfer to Eurofins Genomics (Ebersberg, Germany) for DNA extraction. Qiagen spin column kits in and 96-well settings with proteinase K were used, with initial steps done manually. DNA was returned to SIMPLER and stored at −80 °C in the SIMPLER biobank. Prior to use, the quality of the extracted DNA was assessed using a NanoDrop 1000 spectrophotometer and the quantity measured by the Qubit 4 fluorometer (Thermo Fisher Scientific, Uppsala, Sweden).

#### 2.3.2 DNA sequencing

Full 16S rRNA gene sequencing was done using Oxford Nanopore Technology at the Department of Odontology, Umeå University, Sweden, as previously described (15). Briefly, the v1 through v9 segments were PCR amplified using the primers 27F 5′-AGAGTTTGATCMTGGCTCAG-3′ and 1,492R 5′-CGGTTACCTTGTTACGACTT-3′. Following confirmation of a single fragment of the expected 1,465 bp, amplicons were purified using the 0.4× AMPure XP Beads (Beckman Coulter, Brea, CA, USA), washed with ethanol, and quantified using the Qubit dsDNA HS Assay Kit and Qubit 4.0 fluorometer (Thermo Fisher Scientific, OR, USA). Library preparations were performed by barcoding the amplicons using the Native Barcoding Kit 96 V14 (SQK-NBD114.96) kit (Oxford Nanopore Technologies Nanopore, Oxford, UK). Barcoded samples were pooled and cleaned using 0.4× AMPure XP Beads, and a Native Adapter was fused using T4 DNA Ligase (NEB, E6056). Following additional cleaning with 0.4× AMPure XP Beads (Beckman Coulter), libraries were quantified using a Qubit 4 fluorometer (Thermo Fisher Scientific, OR, USA), loaded on a pre-primed R10.4.1 flow cell (Oxford Nanopore Technologies), and sequenced using a GridION nanopore sequencer (Oxford Nanopore Technologies) for 72 h. Base-calling of nanopore signals and demultiplexing were performed on the GriION using the MinKNOW (Nanopore, Oxford, UK), the Dorado base-callers Super accurate (SUP) model, and Porechop (version 0.2.4, https://github.com/rrwick/porechop), generating demultiplexed FastQ files, with a sequence quality score (QC) of ≥10 and a read length between 1,350 and 1,800 bp.

A commercial mock community (ZymoBIOMICS Microbial Community DNA Standard, D6305, NordicBiosite, Stockholm, Sweden) with pre-purified DNA was used as a positive control, and ultrapure water as a negative control.

#### 2.3.3 Sequence quality control and processing

Raw sequence quality filtering was done with the Emu pipeline (https://github.com/treangenlab/emu) developed for stringent error filtering of Oxford Nanopore Technologies (ONT) sequences (16). The abundance filter was set to >0.0001 [–min-abundance 0.0001]. Retained sequences were classified against the *e*HOMD database (17) pre-built for Emu v3.0+. Samples with >14,900 matching reads were retained. For association analyses, detection prevalence (>0 reads) and relative abundance were calculated for each species. For α- and β-diversity assessments, data were rarefied to the lowest accepted sequencing depth (14,932 reads) using the MicrobiotaProcess package in R studio. For α-diversity, species richness, Shannon index, Simpson index, and Pielou’s scores were estimated. For β-diversity, the Bray–Curtis distance matrix was used.

### 2.4 Statistical analysis

Descriptive dental and microbiota data are presented as means with standard deviation or standard error, or as proportions (%), with group differences tested by parametric or non-parametric tests, respectively. For microbiota analyses, descriptive statistics and regression analyses included species with at least two reads in two or more samples. The MaAsLin3 (Microbiome Multivariable Associations with Linear Models) (18) in R studio (19) was used to determine generalized multivariable linear modeling associations between phenotype (age, number of teeth, and total number reads in the sample) and microbial data across all observations. Taxonomic input consisted of read counts, followed by total sum scaling and log transformation. A nominal false discovery rate (FDR) level of 0.05 was applied if >99 taxa were evaluated for the same outcome. Primary association analyses were restricted to observations with a ≤4-year difference between saliva collection and dental examination. Additional sensitivity analysis for periodontal traits included all possible observations regardless of the time difference between saliva collection and dental examination.

Untargeted machine learning (partial least squares regression, PLS) was employed to select the most influential bacterial species for outcome variation using SIMCA P+ (version 18.0, Sartorius Stedim Data Analytics AB, Umeå, Sweden). Taxa with a vector loading value >|0.1| and a variable importance in the projection (VIP) value >1 were considered the most influential taxa for each outcome, as illustrated in volcano plots and listed in Supplementary Tables S2–S6. PLS modeling in SIMCA+ allows covarying variables and inclusion of covariates but does not explicitly adjust for covariates. Pre-analyses of PLS models without and with covariates (sex, age at saliva collection and dental recording, time window between saliva collection and dental recording, and total read numbers) were conducted, yielding overall similar patterns for ranking of influential species in relation to the tested traits. Therefore, values from models including bacteria only were used for the following analyses. Two part models were fitted which tested effects on both species detection and species abundance (in the subset of observations with non-zero values) (20). Analyses were conducted using the MaAsLin3 pipeline and caution applied if the species were present in less than 50 observations (50 total observations for linear fits, 50 non-zero observations for logistic fits), as recommended by the Huttenhower Lab (18). Therefore, logistic regressions were conducted on binary coded data (detection vs. not), and linear regressions were applied on continuous transformed data for non-zero abundance values. All models included the covariates sex, age at dental examination (dental outcomes) or age at saliva sampling (age outcomes), time window between saliva sampling and dental examination, and total number of reads. Both ages were not included in the same model as they were highly correlated (Pearson correlation coefficient >0.8). *P*-values were FDR corrected if all species were analyzed in all 1,093 samples but not if run for <100 species.

The taxa *S. mutans* and *S. wiggsiae* were specifically noted for caries traits (21, 22), while *A. actinomycetemcomitans, F. alocis, . gingivalis, T. forsythia, T. denticola*, and *T. socranskii* were noted as marker bacteria for periodontal status (3). These species have been repeatedly reported as disease-associated and causally confirmed in experimental settings (23–25).

The software STATA, version 17 (StataCorp LLC, College Station, TX, United States), SIMCA P+ (version 18.0, Sartorius Stedim Data Analytics AB, Umeå, Sweden), and the MasLin3 package (18) in R (26) were used for count transformations, descriptive comparisons, and multivariate and multivariate regression analyses.

## 3 Results

### 3.1 Study group for oral microbiota assessment in aging Swedes

The present study included participants from the SIMPLER cohort (https://www.simpler4health.se/about-us/) with available saliva DNA, questionnaire data, medical information, and dental status. Of 1,118 DNA samples, 1,093 samples yielded >14,900 reads with a mean (min; max) of 58,306 (14,932; 293,486) sequences per sample (Figure 1). Data for these 1,093 participants were used for diversity and profile analyses. Mean sequence read values did not differ between men and women, between younger (<65 years) and older ( ≥65 years) participants, or between those with data on caries or periodontal status.

Most participants were men (93.6%), reflecting that the COSM sub-cohort in SIMPLER primarily recruited men but with the option for a spouse to join. The age at saliva collection ranged from 54 to 84 years. Overweight was common, as indicated by a mean body mass index (BMI) of >26, whereas mean total cholesterol and blood pressure values were normal. Fewer than 6% of the participants were current smokers at the time of saliva collection and 15% had a confirmed diagnosis or signs of diabetes (Table 1).

**Table 1:** Study group demographics for participants with information on caries or periodontal status.

Demographic characteristic	Participants with caries status	Participants with periodontal status
Sex, % men	93.6	93.6
Age at saliva collection, mean (SD)	63.9 (4.9)	64.0 (4.9)
BMI, mean (std)	26.6 (3.8)	26.6 (3.7)
b-Cholesterol, mmoL, mean (SD)	5.2 (1.1)	5.2 (1.1)
Systolic blood pressure, mmHg, mean (SD)	141.0 (15.8)	141.0 (15.9)
Systolic blood pressure, mmHg, mean (SD)	80.1 (7.8)	80.1 (7.8)
Smoking, %	5.8	5.7
Diabetes, %	15.2	15.0
Number of present teeth, mean (SD)	24.6 (4.7)	24.4 (4.7)
Edentulism, *n* (%)	7 (0.6)	7 (0.7)
DMFS, mean (SD)	54.7 (29.9)	—
Any sign of caries (D0 > 0), *n* (%)	1,069 (98.8)	—
Number of D0 surfaces, mean (SD)	72.6 (30.1)	—
Participants with a D2 or D3 caries score, *n* (%)	187 (5.5)	—
Number of D2 or D3 surfaces^a^, mean (SD)	0.31 (0.91)	—
PPD-mean^b^, mean (SD)	—	2.9 (0.44)
Number of teeth with 6 mm PPD, mean (SD)	—	2.8 (3.0)
Participants with at least two 6 mm PPD teeth, *n* (%)	—	598 (55.7)

a Among those with a caries examination within 4 years (*n* = 617), 48 had at least one D3 lesion.

b Average probing pocket depth for present teeth.

All 1,093 participants had information on dental status, with 1,082 having data on caries and 1,073 on periodontal status. Dental information was accumulated between 2 and 14 years after saliva collection, with 40% assessed within 2 years and 57% within 4 years (Figure 1). The study group was characterized by a low prevalence of edentulism (0.6%) and a high number of present teeth (mean 24 teeth, excluding third molars). The teeth were heavily affected by signs of caries or periodontitis. In particular, 43% of the tooth surfaces had an untreated, manifest caries lesion (D3) or caries-related restoration, and 56% of participants had at least two teeth with a probing pocket of 6 mm or deeper combined with a recorded diagnosis or treatment of periodontitis (surgery or scaling/depuration) (Table 1).

### 3.2 Microbial diversity of the oral microbiome

**Figure 2:**
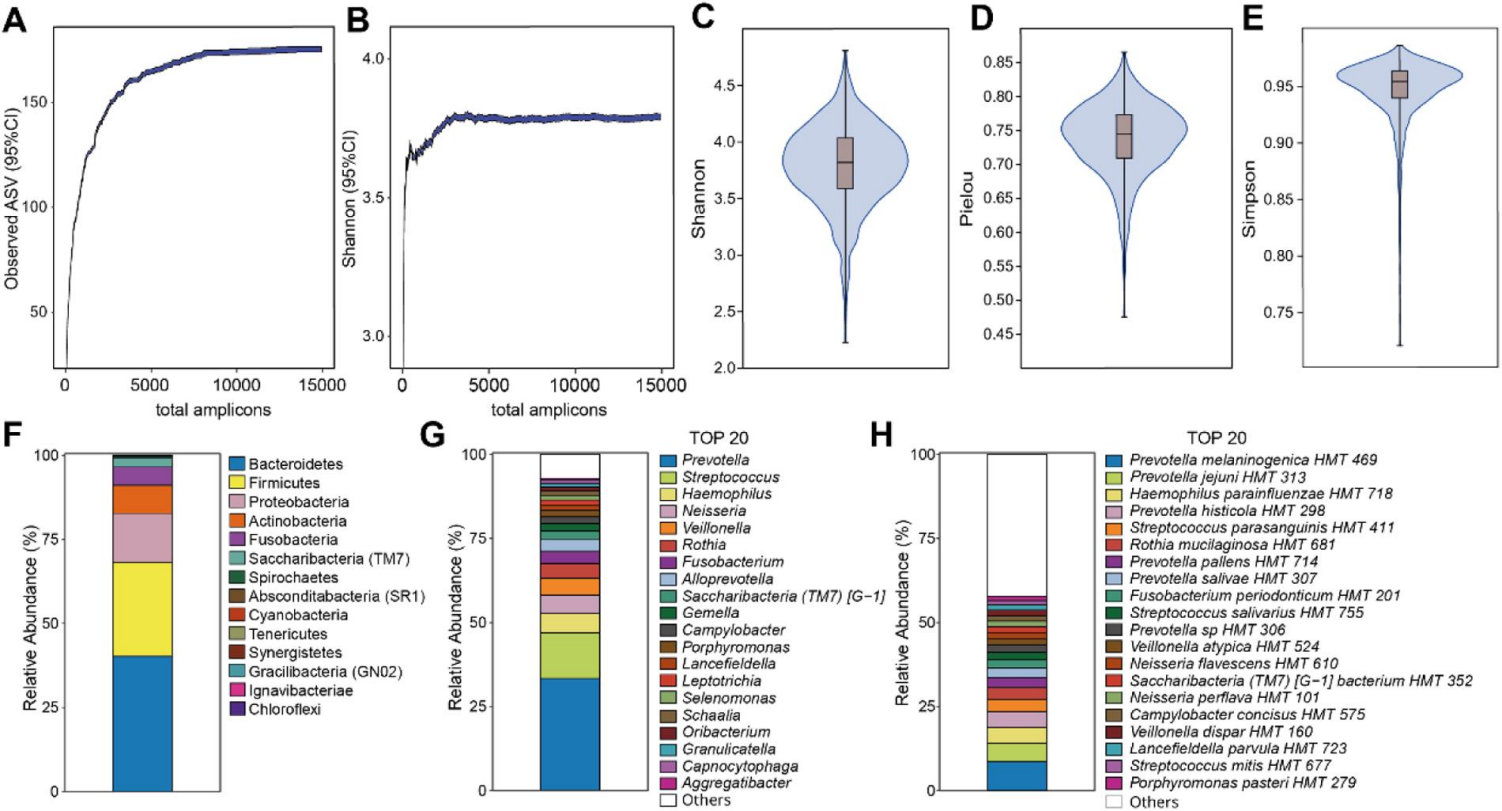
Overview of saliva microbiota profile. The distribution of (**A**) observed bacterial amplicon sequence variants (ASVs) and (**B**) Shannon diversity is presented according to the number of amplicons. Alpha diversity measures, including (**C**) Shannon, (**D**) Pielou, and (**E**) Simpson indices, are depicted at an amplicon depth of 14,932. The composition of (**F**) all phyla, the top 20 (**G**) genera and (**H**) species are illustrated for the 1,093 samples at an amplicon depth of 14,932.

Phylotypes present with at least two reads in at least two participants comprised 580 phylotypes in 78 genera. These represented 260 unnamed phylotypes and 310 named species, among which 6 species were represented by two or more strains (Supplementary Table S1). A core microbiome, defined as present in ≥95% of the participants, included 41 taxa across 13 genera. These were *Streptococcus* (13 species), *Prevotella* (9 species), *Veillonella* (4 species), *Gemella* (3 species), and two species each in *Fusobacterium, Rothia,* and *Schaalia*, plus one species each in *Campylobacter, Granulicatella, Haemophilus, Lancefieldella, Leptotrichia,* and *Oribacterium*. An overview of the diversity and taxonomic profile across the study group is provided in Figure 2, and a full list, including number of reads and relative abundance per species, is provided in Supplementary Table S1.

For reading simplicity, both unnamed phylotypes and named species will hereafter be referred to as species, but with inclusion of the unique human microbiota taxon (HMT) ID for identification of strain from the *e*HOMD database, and relative abundance will be referred to as abundance.

### 3.3 Oral species profile by host and sequencing characteristics

**Figure 3:**
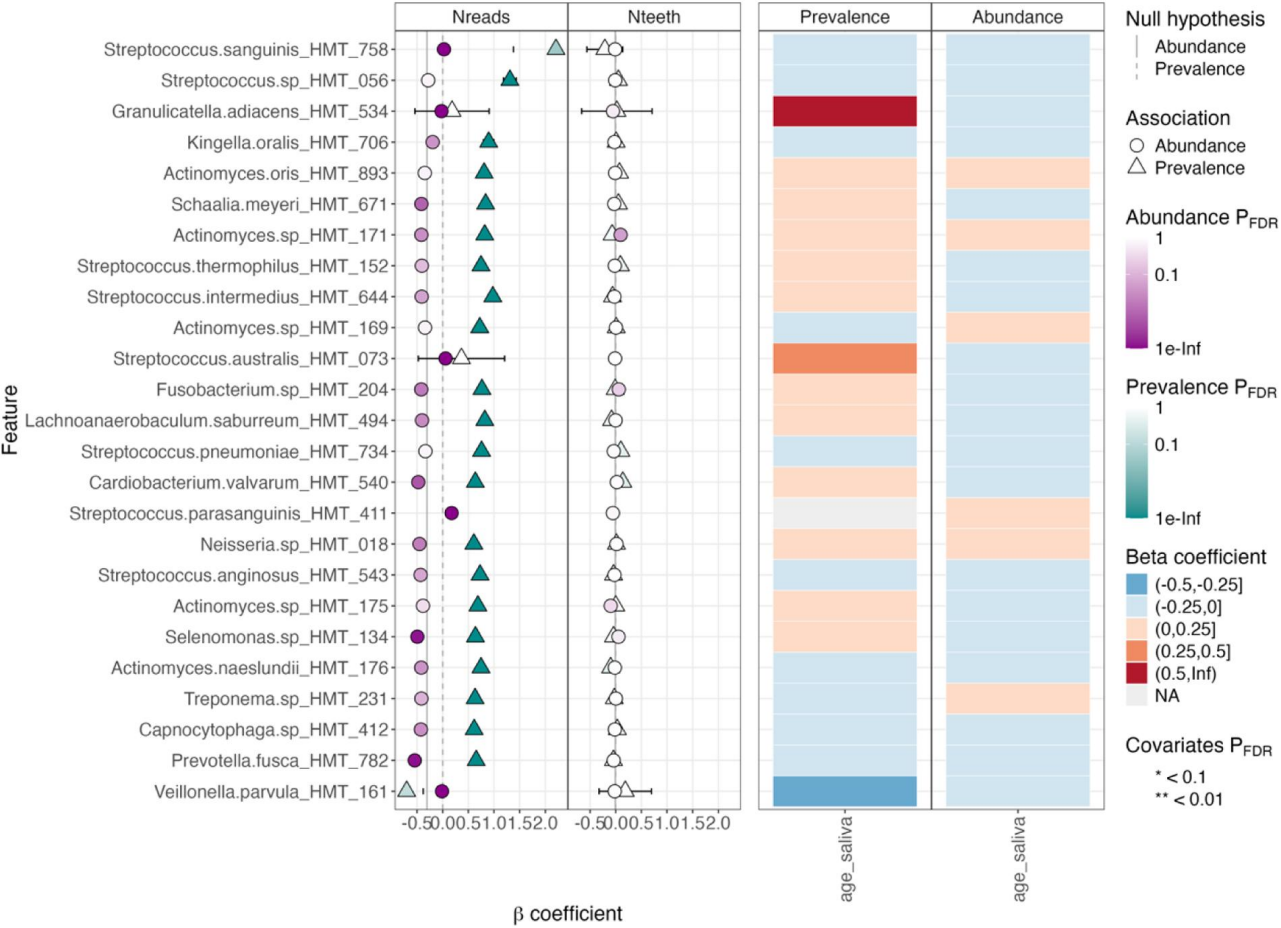
Results from MaAsLin3 regression of species prevalence and relative abundance in those carrying the species. The left panel shows associations with total number of reads for the top species. The middle panel shows associations with number of teeth and the right panels associations with age at saliva collection. Triangles refer to prevalence and circles to abundance and the strengths of associations are color coded as indicate to the right of the panels. For age, FDR-adjusted *p*-values would (if present) have been indicated by stars.

Associations between the total number of sequencing reads, number of teeth, and age at saliva collection were evaluated for the 1,093 samples using the MaAsLin3 package (Figure 3) (18). Due to the low proportion of women, sex differences were not evaluated. As expected, the prevalence (detection) of species was associated with increasing total number of reads for many species. The most notable were in *Streptococcus* (seven species, FDR-adjusted *p*-value < 0.05)—led by *Streptococcus sanguinis* and *Streptococcus sp. HMT056*—and in *Actinomyces* (5 species, FDR-adjusted *p*-value < 0.05) (Figure 3, left panel). Unexpectedly, the abundance of several species was associated with the total number of reads but with mixed directions (Figure 3, left panel). Notably, these associations were observed despite total sum scaling and log transformation of counts. Except for *Actinomyces sp. HMT171* (q < 0.05), no statistically significant association was seen between the number of teeth and species prevalence or abundance (Figure 3, middle panel). Increasing age was associated with increasing prevalence of *Granulicatella adiacens* and *Streptococcus australi*s (β-coefficients >0.5 and >0.25, respectively) and decreasing detection of *Veillonella parvula* (β-coefficient < −0.25), though all with FDR-adjusted *p*-values >0.05. No associations were observed between species abundances and age at saliva collection (β-coefficients ≤ |0.25|) (Figure 3, right panels).

### 3.4 Dental trait and microbiota characteristics by time gaps between saliva collection and dental examination

**Figure 4:**
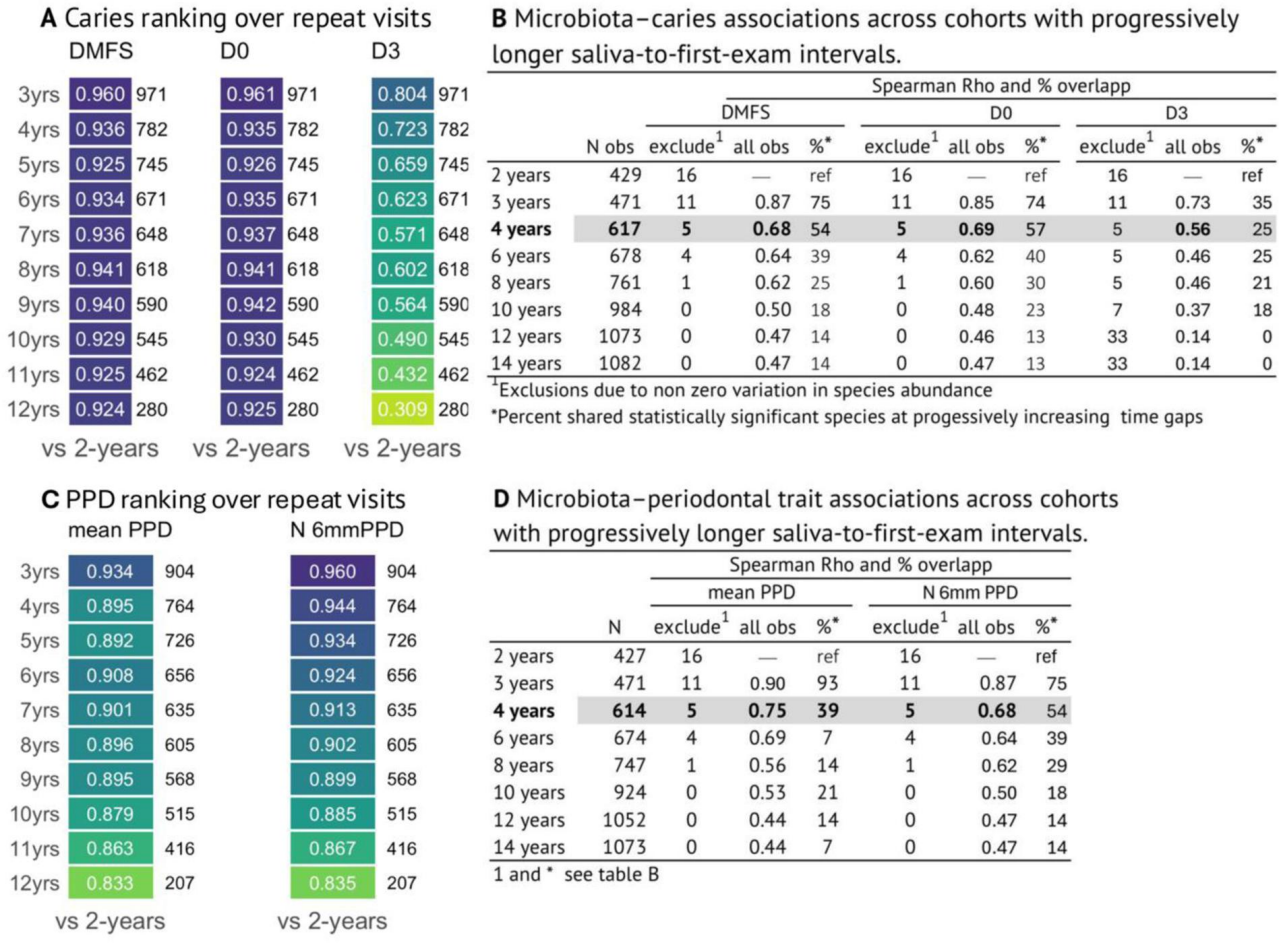
Heat maps for Spearman correlation coefficients between dental scores at the first available dental examination and scores at subsequent annual dental visits for (**A**) caries and (**C**) periodontal traits, and tables showing Spearman correlation coefficients between coefficients from adjusted linear regressions models employing species abundance and dental traits over increasing elapsed times between saliva collection and dental status at the first available examination for (**B**) caries and (**D**) periodontal status.

Two approaches were used to assess the impact of time gap between saliva collection and dental examination. These analyses aimed to help guide decisions about inclusion criteria for association analyses between saliva microbiota dental traits. The first approach evaluated correlations between dental traits at first available and follow-up examinations, demonstrating high stability in these traits during the first few years of follow-up (Figures 4A,C). The second approach fitted regression models between microbiota and dental traits repeatedly, while increasing the cohort size by allowing progressively wider time gaps for the first available examination from 2 to 14 years (Figures 4B,D). These analyses revealed shifts in association patterns when including dental observations many years after saliva collection. Based on these findings, we included a time gap of up to 4 years as a pragmatic trade-off between statistical power and measurement accuracy.

### 3.5 Associations between species profiles and caries traits

**Figure 5:**
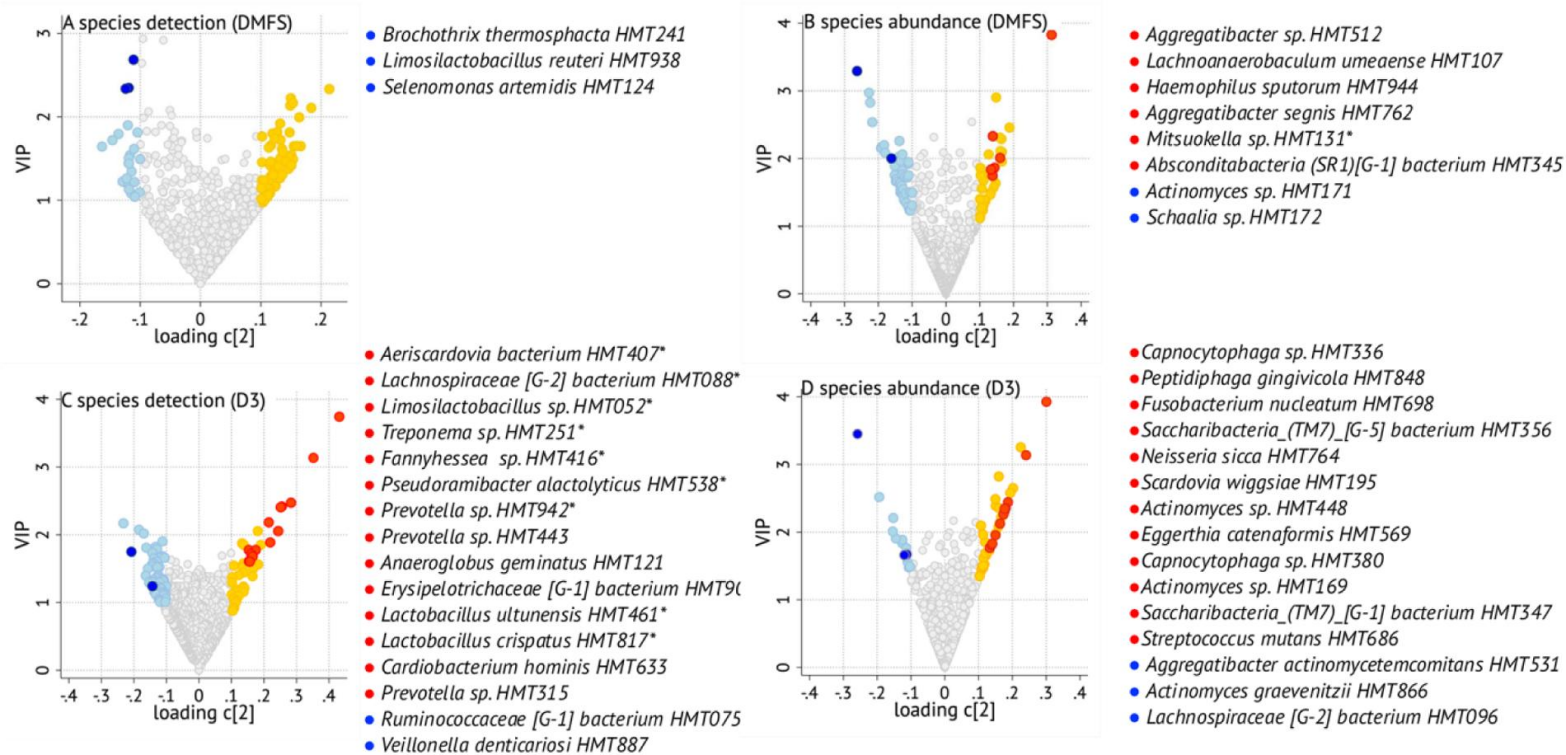
Volcano plot illustrating associations between saliva species and DMFS (**A,B**) or D3 (**C,D**). The figures are based on *n* = 617 participants with up to 4-year difference between saliva collection and dental scoring, with right arms showing species positively (more disease) associated and the left arms negatively associated taxa (the full list is found in Supplementary Tables S2, S3) with the respective outcome. Red and dark blue dots refer to species indicated by PLS (a loading on component 2 > |0.1| and VIP > 1.0) and remaining significant in covariate-adjusted regression; yellow and light blue dots are species indicated by PLS but not supported in regression; and gray dots are species that were not found influential in PLS. Asterisks refer to species present in <50 samples.

Associations between taxa prevalence and abundance and the caries traits DMFS (reflecting life course caries experience in permanent teeth) and number of manifest caries lesions (D3) were evaluated using PLS in participants with four or fewer years between saliva sampling and dental examination (*n* = 617). Volcano plots displaying the top 10 species from PLS (or more if the number of statistically significant species exceeded 10 or the marker species *S. mutans* and *S. wiggsiae* were found) are provided in Figure 5. Complete lists of species with a loading on component 2 >|0.1| and VIP >1.0, i.e., indicated as influential for the caries trait variation, and forwarded to covariate-adjusted regression, are provided in Supplementary Tables S2, S3.

*Species detection and DMFS scores:* PLS identified the presence of 59 species (31% present in <50 samples) with a loading on component 2 >|0.1| and VIP >1.0 as associated with high DMFS values (Supplementary Table S2). These included the cariogenic *Streptococcus sobrinus* as well as the periodontal pathogens *F. alocis* and *T. forsythia* (Figure 5A). However, none was statistically significant in covariate-adjusted MaAsLin3 regression*.* By the same criteria, 24 species (21% present in <50 samples) were indicated by PLS as associated with low DMFS scores (Supplementary Table S2)*.* Three of these reached statistical significance in MaAsLin3 modeling with covariate adjustment, i.e., *Brochothrix thermosphacta,* a food spoilage species, and *Limosilactobacillus reuteri*, both present in ≤10 samples, leaving *Selenomonas artemidis* as the sole solid candidate.

*Species abundance and DMFS scores:* PLS indicated 34 species (9% present in <50 samples) as influential for high DMFS scores (Supplementary Table S2). Of these, covariate-adjusted regression identified *Aggregatibacter sp. HMT512, Lachnoanaerobaculum umeaense, Haemophilus sputorum, Aggregatibacter segnis, Mitsuokella sp. HMT131,* and *Absconditabacteria (SR1)[G-1] bacterium HMT345*) as significant (Figure 5B). High abundance of 42 species (2% present in <50 samples) was indicated by PLS as associated with low DMFS scores, with *Actinomyces sp. HMT171* and *Schaalia sp. HMT172* significant in the adjusted regression (Supplementary Table S2). Neither the presence nor abundance of *S. mutans* or *S. wiggsiae* was indicated by PLS as influential for DMFS variation. The lack of association was confirmed in covariate-adjusted regressions (*p*-values >0.05).

*Species detection and manifest caries lesions (D3)**:*** Applying the criteria described earlier, PLS identified the presence of 42 species (64% present in <50 samples) as associated with having high numbers of manifest caries lesions (D3), with 14 also being statistically significant in the covariate-adjusted regression, including *Lactobacillus ultunensis* and *Lactobacillus crispatus* (Supplementary Table S3, Figure 5C). Notably, only *Anaeroglobus geminatus*, *Cardiobacterium hominis*, *Prevotella sp. HMT443*, and *Prevotella sp. HMT315*) were present in more than 50 samples (Supplementary Table S3). PLS also indicated that few D3 surfaces were associated with detection of 59 species (31% present in <50 samples), of which *Ruminococcaceae [G-1] bacterium HMT075* and *Veillonella denticariosi* were statistically significant in the adjusted regression (Figure 5C, left arm).

*Species abundance and manifest caries lesions (D3)**:*** PLS indicated 40 species (3% present in <50 samples) as positively associated with high numbers of D3 tooth surfaces, with 12 of these in covariate-adjusted regression (Figure 5D, Supplementary Table S3). These included *S. wiggsiae and S. mutans* abundances. In addition, 14 (0% present in <50 samples) species were indicated by PLS as associated with few D3 surfaces, with three [*A. actinomycetemcomitans, Actinomyces graevenitzii,* and *Lachnospiraceae (G-2) bacterium* HMT096] being significant in covariate-adjusted regression too. Among the remaining nine species, the generally health-associated *S. sanguinis* was observed, as well as the periodontal pathogen *P. gingivalis.*

### 3.6 Associations between species profiles and periodontal traits

**Figure 6:**
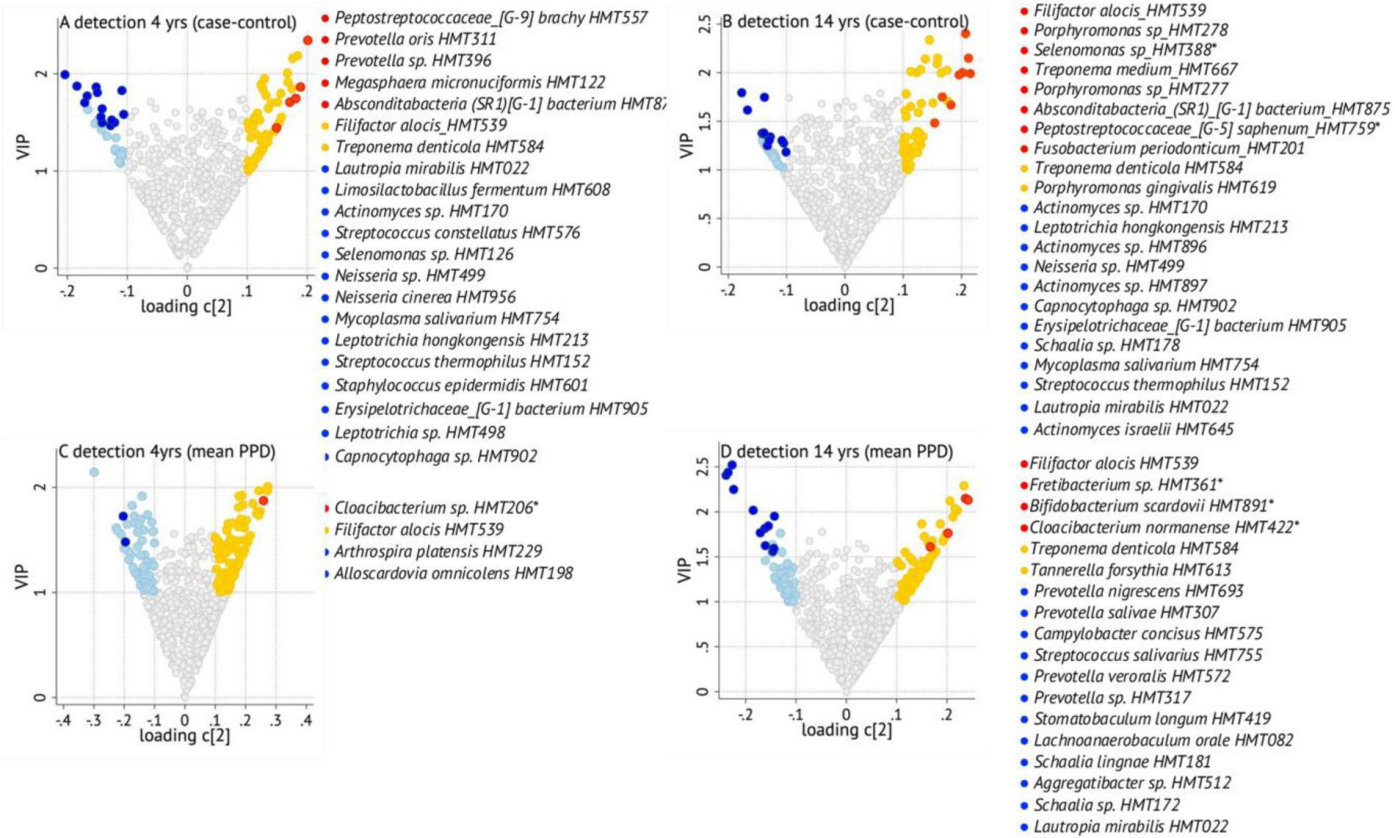
Volcano plot illustrating associations between saliva species and having two or more 6-mm-deep probing pockets or not (case vs. control) (**A,B**) or mean probing pocket depth (mean PPD) (**C,D**). The figures are based on *n* = 614 participants with up to 4-year (**A,C**) or *n* = 1,073 up to 14-year (**B,D**) difference between saliva collection and dental scoring, with right arms showing species positively associated with poor status, and the left arms negatively associated (the full list is found in Supplementary Tables S4, S5) with the respective outcome. Red and dark blue dots refer to species indicated by PLS (a loading on component 2 > |0.1| and VIP > 1.0) and remaining significant in covariate-adjusted regression; yellow and light blue dots are species indicated by PLS but not supported in regression; and gray dots are species that were not found influential in PLS. Asterisks refer to species present in <50 samples.

Models were run with four or fewer years between sampling and dental examination (*n* = 614) as primary analyses, and for all participants with periodontal data regardless of time differences (*n* = 1,073) as sensitivity analyses. Two outcomes for periodontal status were tested: (1) a binary outcome from having at least two teeth with a 6-mm pocket (cases) or not (controls) and (2) the mean of PPD for all probed sites. The marker species based on existing literature were *A. actinomycetemcomitans, F. alocis, P. gingivalis, T. forsythia, T. denticola,* and *T. socranskii.* Similar to caries, volcano plots showing top 10 species from PLS, with eventual additions as described earlier, are shown in Figure 6. Complete lists of species with a loading on component 2 >|0.1| and VIP >1.0, and forwarded to covariate-adjusted regression, are found in Supplementary Tables S4–S6.

*Species detection and having at least two teeth with a 6 mm PPD:* Primary PLS analysis indicated 68 species as associated with being a case in the up-to-4-year window and 59 in the up-to-14-year window (36% in <50 samples) (Supplementary Table S4). Of these *F. alocis* and *T. denticola* were indicated in both time windows, while *P. gingivalis* was indicated in the longer time window (Figures 6A,B)*. Filifactor alocis* in the up-to-14-year window was the only species that remained statistically significant when including covariate*s* (Figures 6A,B)*.* The primary analysis indicated 30 species as associated with being in the control group in the up-to-4-year window (53% < 50 samples), while the sensitivity analysis identified 34 species in the up-to-14-year window (62% < 50 samples). In both time windows, 14 and 13 species, respectively, remained statistically significant in the covariate-adjusted regressions. None of these represented a periodontitis or caries marker species (Supplementary Table S4).

*Species detection* and *mean probing pocket depth:* PLS identified 89 species (52% < 50 samples) in the up-to-4-year window and 70 species (44% < 50 samples) in the up-to-14-year window to be associated with a higher mean PPD (Supplementary Table S5). Similar to the case–control setting, the longer time window (compared to the shorter) identified more association with the periodontitis marker bacteria. Thus, *F. alocis, T. denticola*, and *T. forsythia* were included, with *F. alocis* remaining in the covariate-adjusted regression (Figure 6C). Notably, *Cloacibacterium normanense* was significant in up-to-4-year window and suggested by PLS in the longer time window (Figure 6D). Further, PLS identified 50 species (44% < 50 samples) in the shorter time window and 51 species (36% < 50 samples) in the longer time window to be associated with a lower mean PPD.

*Species abundance and periodontal status:* Apart from a higher abundance of *T. forsythia* in cases identified by PLS or covariate-adjusted regressions, none of the marker species were identified as influential for case vs. control status or for high mean PPD in the shorter and longer time windows (Supplementary Figure S1, Supplementary Table S6). Notably, a positive association, similar to that found between *Cloacibacterium sp. HMT206* prevalence and mean PPD, was seen with its abundance and being a case and having high mean PPD.

**Figure 7:**
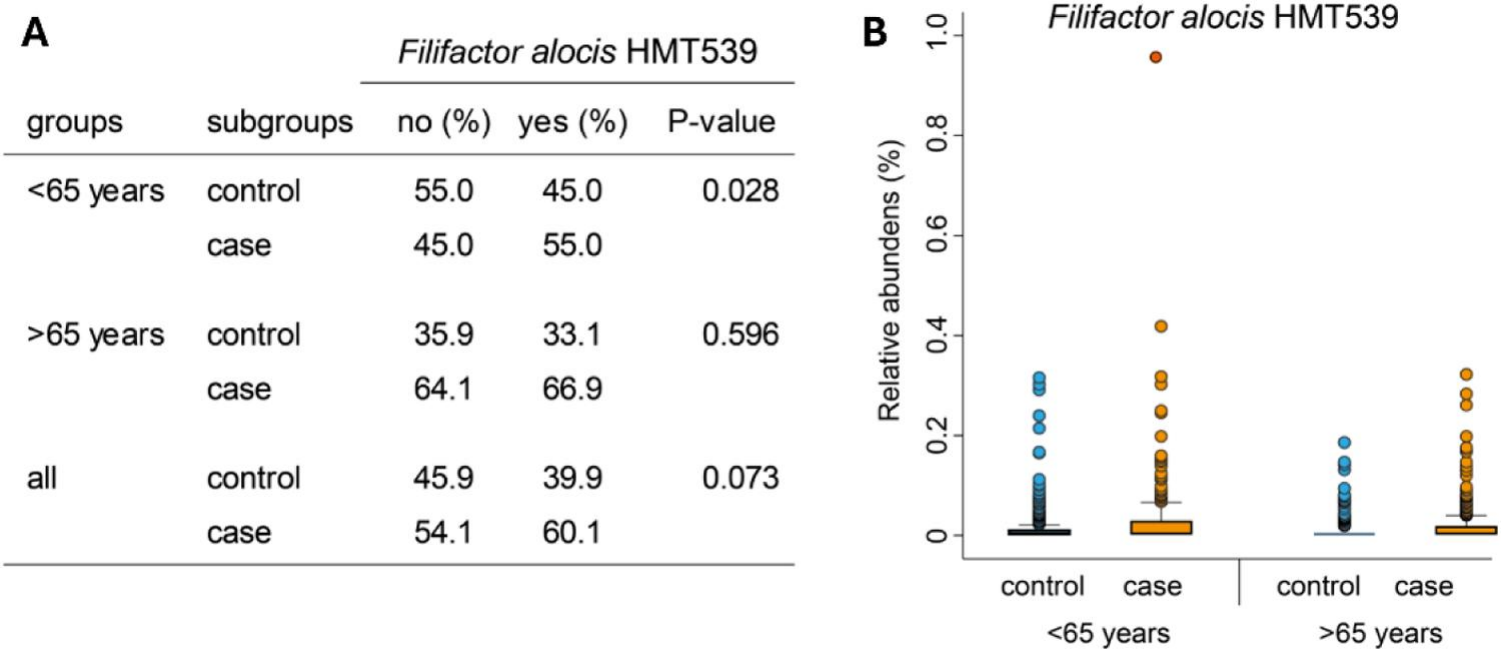
Associations between *F. alocis* presence or relative abundance and periodontal status. (**A**) Cross tabulation of being a case or control by the criteria defined in the present study versus having *F. alocis* or not in those being <65 years at saliva collection or ≥65 years and for all together. (**B**) Box plots of the relative abundance by age groups defined in (**A**).

*Filifactor alocis and periodontal status:* Given the consistent association between the presence and abundance of *F. alocis* with periodontitis case status under the criteria defined here, we evaluated this in more detail. There was a tendency toward a higher prevalence of cases carrying *F. alocis* compared to controls (*p* = 0.073), which was evident among participants younger than 65 years (*p* = 0.028, Figure 7A). The trend was similar when examining relative abundances (Figure 7B).

## 4 Discussion

This study examined the oral microbiota in an aging Swedish population, characterized by a high retention of natural teeth, extensive dental restorations, and prevalent signs of active disease. With increasing life expectancy and advancements in dental care, such dental profiles are becoming more common globally. Concurrent age-related changes in health conditions and medication use necessitate specialized knowledge tailored to these demographics. In this context, we explored the oral microbiota and assessed the performance of different measures capturing caries and periodontal disease. Our findings indicated marked individual variations in oral microbiota but with no significant age-related trends in diversity or composition across the age range of 54–84 years. However, in addition to highlighting previously untracked species, we observed enrichment of species typically associated with caries or periodontal disease in individuals with active disease. Traditional scores reflecting lifelong disease accumulation, especially DMFS for caries, proved to be insensitive in this demographic group.

There is inherent complexity in carrying out molecular characterization of the microbiota, which arises from challenges in taxonomic annotation, variations in read length and sequencing errors, a high prevalence of zero detections, and strong correlations between sample read number and detection depth. Several methods have been suggested to address these challenges, each with its own advantages and disadvantages. In this study, we characterized the saliva microbiota by targeting the full 16S rRNA gene, which provides superior taxonomic resolution compared to using one or two variable region amplicons, while maintaining reasonable costs. This method also circumvents problems arising from the high proportion of human DNA in saliva extractions (as compared to feces and shotgun sequencing) through targeted PCR amplification (15). To mitigate biases associated with read number and detection depth, we utilized rarified data for diversity analyses and applied total sum scaling followed by a log transformation, including the transformed values as covariates in the regression. The impact of read numbers, even after scaling, was especially strong for detection of prevalent species in the *Streptococcus* and *Actinomyces* genera, underscoring the importance of including total number of reads as covariate in regression analyses, as emphasized by the Huttenhower group (18). In addition, we applied relatively relaxed criteria for PLS identification. Typically, a VIP value of 1.0 is considered influential for outcome variation, while a value of 1.5 is highly influential. Our choice aimed to balance the need to limit the number of species tested in the follow-up regressions against the risk of missing influential taxa. For the follow-up sensitivity regressions, a two-step model was employed to distinguish the impact of carrying a species from the impact of its abundance levels when present, addressing the challenge of high numbers of zero values as applied in the MaAsLin3 package.

The present study revealed that in countries like Sweden, the aging population rarely experiences complete tooth loss, yet their dental status commonly shows extensive signs of lifelong caries and periodontal disease. For caries, the DMFS score commonly reflects lifelong disease burden, and therefore our initial evaluation targeted DMFS. However, this failed to detect any association with the marker species *S. mutans* and *S. wiggsiae* but highlighted several periodontitis-associated species, especially *F. alocis*. The “missing” component in DMFS should represent loss due to caries; however, surfaces missing due to other reasons are also captured. While this may be useful in younger groups, in populations like the present, the inclusion of missing surfaces in the score can falsely inflate the score due to teeth lost to periodontal disease, which may be the case here. Therefore, we evaluated the association with a phenotype targeting present disease and excluding both the missing and filled components, i.e., untreated caries cavities (D3). Indeed, D3 was associated with cariogenic bacteria such as *S. mutans* and *S. wiggsiae*. The discrepancy between DMFS and D3 may stem from either of these two components, since each surface status is associated with its own intrinsic prerequisite and explanation. Decayed surfaces represent a unique niche characterized by low pH compared to filled surfaces. The microbiota in deep caries lesions has been reported to be dominated by lactobacilli with a relative abundance up to 42% (27). Notably, detection, but not abundance, of two lactobacilli species was associated with high D3 in the PLS and MaAsLin3 regressions. Thus, while the discrepancy might involve a biological difference between the two phenotypes, the abundance of lactobacilli around 0.2% suggests that in this population the seemingly better behavior of D3 vs. DMFS is not mainly a biological effect, but rather due to inflation from the missing or filled component. This may, however, differ in other demographics. In contrast, both proxies used for periodontal status, i.e., having two or more teeth with a 6-mm-deep gingival pocket or mean probing pocket depth, were associated with the expected marker species.

The present analyses highlighted several significant associations, but comparisons with previous studies in the aging population are hampered by different approaches in sequencing, bioinformatics, and the fact that most prior studies have been small or targeted selected groups, such as individuals with diabetics or frailty. Nevertheless, our findings of associations between *S. mutans, S. sobrinus,* and *S. wiggsiae* with caries, and *F. alocis, T. denticola, T. forsythia*, *A. actinomycetemcomitans,* and *P. gingivalis* with periodontal phenotypes, align with previous studies. This study generated some noteworthy results beyond taxa identifications. Caries phenotypes were mainly associated with species abundances, whereas periodontal phenotypes were associated with species detection. One possible interpretation is that host factors favoring colonization play an important role in periodontitis, whereas environmental conditions favoring thriving are important for caries-associated traits, such as sugar-induced pH drop and *S. mutans* enumeration (28). A further notable finding was the stability of associations between microbiota profiles and periodontal traits across the shorter (4 years) and longer (up to 14 years) time windows. This supports stability in the microbiota in upper age groups of periodontally compromised adults, but given the compromising effect of the F or M components, this concept is not accountable for DMFS. Another notable finding was that species present in few samples (<50 individuals) were more frequently associated with disease, whereas species associated with low disease scores were more abundant. This may be an illustration of disease dysbiosis, where very low-abundance opportunistic species enumerate but remain at an overall quite low prevalence and abundance. Although this finding needs to be confirmed, it underlines the need for large study groups to achieve power.

The strengths of the present study include its population-based recruitment (although skewed toward male participants) and its considerably larger sample size compared to previous studies of aging populations. Moreover, the study benefited from access to clinically registered dental status from a comprehensive national register and full 16S rDNA sequencing alongside a curated oral species-specific database (15). This approach significantly enhanced taxonomic specificity compared to earlier studies that focused on limited regions of the 16S rRNA gene. There are, however, some limitations that merit attention. First, the dental examinations were performed by multiple dentists who adhered to the baseline standards set by the Swedish Board of Health and Welfare but without additional calibration. In a previous validation study, there was high concordance between caries scores from routine dental visits and recall examination performed by a study examiner (29), suggesting that different Swedish dentists apply similar diagnostic criteria when assessing caries. If present, variation in diagnostic criteria between dentists is not anticipated to correlate with microbiota traits, so this would represent a source of random error, reducing statistical power rather than creating any systematic bias in results. Microbiota sequencing was based on a saliva sampling, meaning it is not possible to comment on other oral microbiotas such as those within a periodontal pocket. In addition, while bone level or attachment loss assessments are considered the gold standard for periodontal health evaluation, the dental register data were confined to probing pocket depths and diagnostic and treatment codes; therefore, the present study used scores as proxies for periodontal disease. This may lead to underestimation of severity of periodontal conditions or misclassification between health and disease (essentially, random noise), which could potentially reduce statistical power or underestimate the strength of association between periodontal status and microbiota traits. However, this would not create spurious associations. The use of periodontal proxy scores means results are not directly comparable to studies employing clinical definitions of periodontitis. Nonetheless, the identification of anaerobic species that have been identified as associated with periodontitis in both clinical and experimental studies supports the use of the selected proxies, although large study groups are needed to balance the noise from misclassifications. It should also be noted that there was a time window of 2–14 years between saliva collection and dental examinations. To mitigate this, we limited the primary analyses to 2–4 years as substantiated by our prior validation of SKaPa-derived caries data (29). Sensitivity analysis across all participants for periodontal outcomes yielded largely consistent results. Further, the predominance of male participants limits generalizability and may affect external validity, underscoring the need to confirm these findings in women.

In conclusion, the present study highlights the importance of carefully considering which dental phenotype to employ in demographics like the one used here. Our findings confirm the role of previously identified caries- and periodontitis-associated species, while also suggesting several more species, such as in the *Fusobacterium* and *Treponema genera*.

## Data Availability

The datasets presented in this article are not readily available because original research data cannot be shared due to GDPR restrictions unless specific ethical and other approvals are obtained. Detailed summary data are shared in the supplementary tables. Requests to access the datasets should be directed to https://www.simpler4health.se/w/sh/en/researchers/data-access.
