# Supplementary material for: Oral microbiota in an aging Swedish population with high dental disease burden: an observational registry-based study
Source: Front Oral Health. 2026 Jan 6;6:1709163. doi: 10.3389/froh.2025.1709163 (PMC12816219; doi:10.3389/froh.2025.1709163)

**Figure S1**. Volcano plot illustrating associations between saliva species abundanc and having two or more 6 mm deep probing pockets (cases) or not (controls) (a,b) or mean probing pocket depth (mean PPD) (c,d). The right arms show species positively associated with taxonomic annotation and the left arms negatively associated taxa (annotations found in Tables S7) with the respective outcome. Red and dark blue dots refer to species indicated by PLS (a loading on component 2 >|0.1| and VIP>1.0) and remaining significant in covariate adjusted regression, yellow and light blue dots are species indicated by PLS but not supported in regression, and grey dots species that were not found influential in PLS. Stars (*) refer to species present in <50 samples


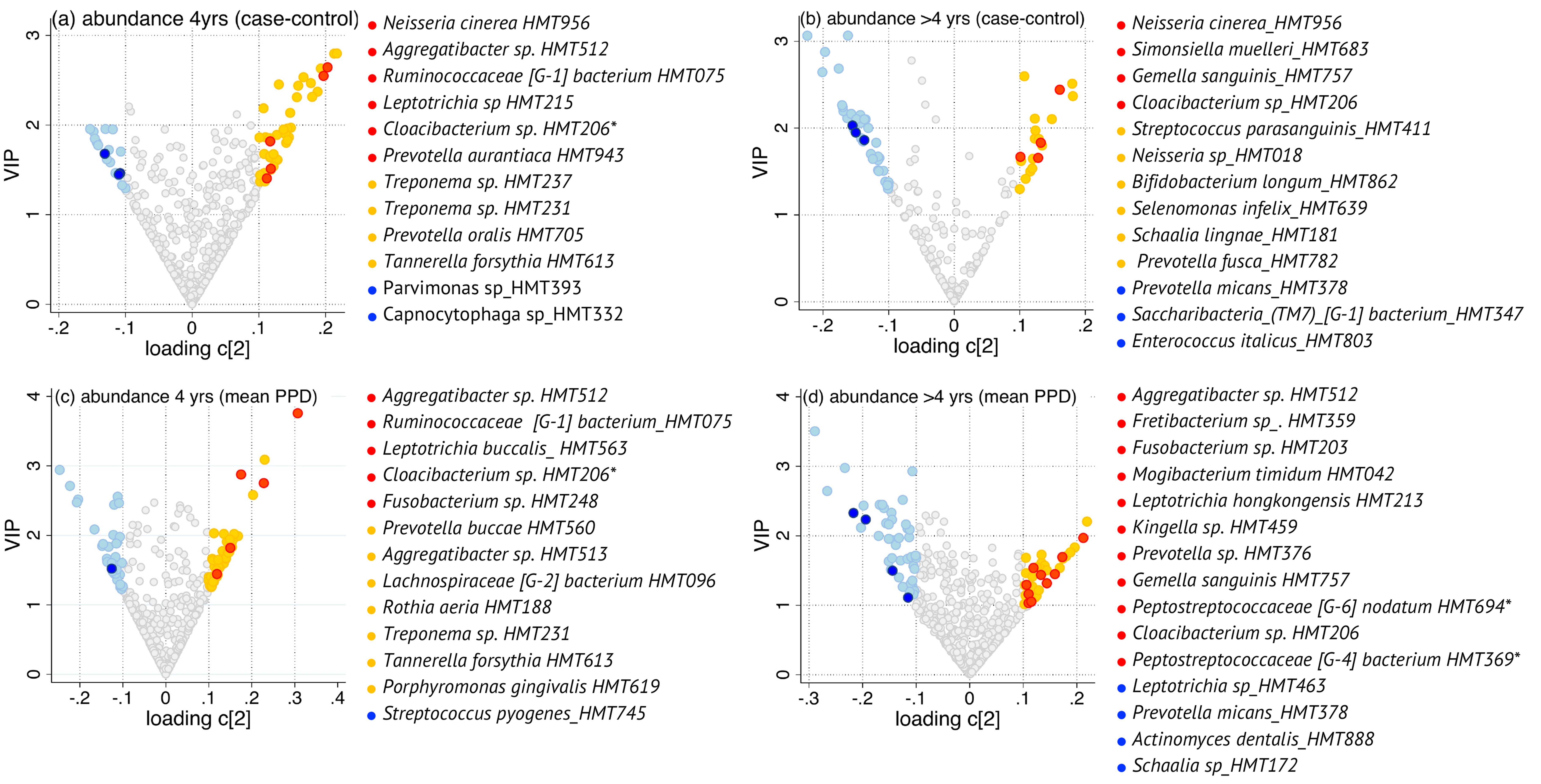

Supplement: Supplementary file 7 [file Supplementaryfile1.docx]
