# Supplementary material for: Oral microbiota in an aging Swedish population with high dental disease burden: an observational registry-based study
Source: Front Oral Health. 2026 Jan 6;6:1709163. doi: 10.3389/froh.2025.1709163 (PMC12816219; doi:10.3389/froh.2025.1709163)
Supplement: Supplementary file 8 [file Supplementaryfile2.doc]

STROBE Statement—Checklist of items that should be included in reports of ***cross-sectional studies***

|  | Item No | Recommendation |
| --- | --- | --- |
| **Title and abstract** | 1 | (*a*) Indicate the study’s design with a commonly used term in the title or the abstract  Described as “observational, registry-based study” in the title and abstract |
|  |  | (*b*) Provide in the abstract an informative and balanced summary of what was done and what was found  See abstract |
| Introduction | | |
| Background/rationale | 2 | Explain the scientific background and rationale for the investigation being reported  Introduction lines 52-57 |
| Objectives | 3 | State specific objectives, including any prespecified hypotheses  Introduction lines 58-61 |
| Methods | | |
| Study design | 4 | Present key elements of study design early in the paper  Methods section 2.1, 2.2, 2.3 and 2.4 |
| Setting | 5 | Describe the setting, locations, and relevant dates, including periods of recruitment, exposure, follow-up, and data collection  Methods section 2.1 |
| Participants | 6 | (*a*) Give the eligibility criteria, and the sources and methods of selection of participants  Methods 2.1 |
| Variables | 7 | Clearly define all outcomes, exposures, predictors, potential confounders, and effect modifiers. Give diagnostic criteria, if applicable  Methods 2.2 and 2.3 |
| Data sources/ measurement | 8* | For each variable of interest, give sources of data and details of methods of assessment (measurement). Describe comparability of assessment methods if there is more than one group  Methods 2.3 |
| Bias | 9 | Describe any efforts to address potential sources of bias  Sequence quality control 2.3.3 |
| Study size | 10 | Explain how the study size was arrived at  NA – all available data were used |
| Quantitative variables | 11 | Explain how quantitative variables were handled in the analyses. If applicable, describe which groupings were chosen and why  Periodontal health groupings section 2.2 paragraph 3 |
| Statistical methods | 12 | (*a*) Describe all statistical methods, including those used to control for confounding  Methods 2.4 |
|  |  | (*b*) Describe any methods used to examine subgroups and interactions  No subgroup analysis |
|  |  | (*c*) Explain how missing data were addressed  Participants with missing dental or microbiota data were excluded – see section 2.1 and flow chart figure 1. |
|  |  | (*d*) If applicable, describe analytical methods taking account of sampling strategy  NA |
|  |  | (*e*) Describe any sensitivity analyses  Section 2.2 paragraph 3, section 2.4 paragraph 1. |
| Results | | |
| Participants | 13* | (a) Report numbers of individuals at each stage of study—eg numbers potentially eligible, examined for eligibility, confirmed eligible, included in the study, completing follow-up, and analysed  Section 2.1 |
|  |  | (b) Give reasons for non-participation at each stage  Section 2.1 |
|  |  | (c) Consider use of a flow diagram  Figure 1 |
| Descriptive data | 14* | (a) Give characteristics of study participants (eg demographic, clinical, social) and information on exposures and potential confounders  Results 3.1 and Table 1 |
|  |  | (b) Indicate number of participants with missing data for each variable of interest  NA – inclusion criteria mean that only participants with complete data were eligible |
| Outcome data | 15* | Report numbers of outcome events or summary measures  Results 3.2 and Figure 2 |
| Main results | 16 | (*a*) Give unadjusted estimates and, if applicable, confounder-adjusted estimates and their precision (eg, 95% confidence interval). Make clear which confounders were adjusted for and why they were included  Tables S2-S6 including standard errors for all tests |
|  |  | (*b*) Report category boundaries when continuous variables were categorized  NA |
|  |  | (*c*) If relevant, consider translating estimates of relative risk into absolute risk for a meaningful time period  NA |
| Other analyses | 17 | Report other analyses done—eg analyses of subgroups and interactions, and sensitivity analyses  Sensitivity analysis reported in right hand portion of tables S2-S6 |
| Discussion | | |
| Key results | 18 | Summarise key results with reference to study objectives  Discussion paragraph 1 |
| Limitations | 19 | Discuss limitations of the study, taking into account sources of potential bias or imprecision. Discuss both direction and magnitude of any potential bias  Discussion paragraph 5 |
| Interpretation | 20 | Give a cautious overall interpretation of results considering objectives, limitations, multiplicity of analyses, results from similar studies, and other relevant evidence  Discussion paragraphs 2,3,4 |
| Generalisability | 21 | Discuss the generalisability (external validity) of the study results  Discussion paragraph 5 |
| Other information | | |
| Funding | 22 | Give the source of funding and the role of the funders for the present study and, if applicable, for the original study on which the present article is based  Funding statement |

*Give information separately for exposed and unexposed groups.

**Note:** An Explanation and Elaboration article discusses each checklist item and gives methodological background and published examples of transparent reporting. The STROBE checklist is best used in conjunction with this article (freely available on the Web sites of PLoS Medicine at http://www.plosmedicine.org/, Annals of Internal Medicine at http://www.annals.org/, and Epidemiology at http://www.epidem.com/). Information on the STROBE Initiative is available at www.strobe-statement.org.
